# Traumatic corneal wound (microns away from a full-thickness laceration)

**DOI:** 10.1093/omcr/omac043

**Published:** 2022-05-23

**Authors:** Shaina N Kumar

**Affiliations:** Department of Ophthalmology, Bronxcare Health System, Bronx, NY, USA

**Figure 1:**
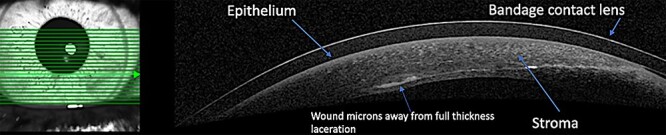
An anterior segment OCT scan reveals the tangential partial-thickness corneal laceration, which passes through the corneal epithelium and stroma; it misses the endothelium by microns, thus preventing a full thickness corneal perforation.

A 12-year-old male presented to the emergency department with right eye pain after being hit with a bungee cord. His visual acuity was 20/20 in the affected eye. His pupillary exam was unremarkable without an obvious afferent pupillary defect. His intraocular pressures were 15 and 19 mmHg (right and left eyes, respectively). Slit lamp exam of the right eye revealed a corneal epithelial defect with an associated stromal defect. Although Seidel testing initially revealed a possible small aqueous leak near the wound apex, this was not appreciated ~1 hour later. Anterior chamber depths were relatively similar between both eyes. The patient was diagnosed with a partial thickness corneal wound of the right eye and Moxifloxacin ophthalmic solution was initiated. A bandage contact lens (BCL) was also placed over the right cornea. An anterior segment optical coherence tomography (AS-OCT) (OCT; Heidelberg Spectralis®) revealed the tangential partial thickness laceration Figure 1). The patient healed well with the continuation of Moxifloxacin. Treatment varies based on wound characteristics for partial-thickness corneal lacerations. A pressure patch and topical antibiotics may suffice if wound edges are well apposed. The advantages of using a BCL are to protect the wound against eyelid movement and promote re-epithelialization. A BCL may also keep patients from needing sutures, which is particularly beneficial for central wounds obscuring the visual axis [1]. Anterior segment OCT offers noninvasive, high-resolution imaging of structures such as the cornea. This method may be especially ideal in the pediatric population in which evaluation of the eye for traumatic injuries can be challenging [2]. Traumatized ocular tissue may likely be fragile and unsuitable for direct contact, again supporting the use of AS denotes Anterior segment-OCT in such scenarios [3]. Similar cases of near-perforating injuries have been reported to benefit from evaluation with AS-OCT [2].
